# Quality of Education and Late-Life Cognitive Function in a Population-Based Sample From Puerto Rico

**DOI:** 10.1093/geroni/igab016

**Published:** 2021-05-09

**Authors:** Cheyanne Barba, Alberto Garcia, Olivio J Clay, Virginia G Wadley, Ross Andel, Ana Luisa Dávila, Michael Crowe

**Affiliations:** 1 Department of Psychology, University of Alabama at Birmingham, USA; 2 School of Public Health, University of Puerto Rico, San Juan, Puerto Rico; 3 Division of Gerontology, Geriatrics, and Palliative Care, University of Alabama at Birmingham, USA; 4 School of Aging Studies, University of South Florida, Tampa, USA

**Keywords:** Cognition, Cognitive reserve, Minority and diverse populations

## Abstract

**Background and Objectives:**

We examined quality of education, literacy, and years of education in relation to late-life cognitive function and decline in older Puerto Ricans.

**Research Design and Methods:**

Our sample consisted of 3,385 community-dwelling adults aged 60 years and older from the Puerto Rican Elderly: Health Conditions study. Quality of education was based on principal component analysis of variables gathered from Department of Education and Census reports. Literacy (yes/no) and years of education were self-reported. Cognitive function was assessed in participants’ homes at baseline and 4 years later using a previously validated Spanish-language 20-point global screening measure for dementia, the minimental Cabán. Regression models were adjusted for sociodemographic and life course covariates.

**Results:**

Quality of education was positively correlated with both educational attainment and cognitive performance. Independent of years of education, literacy, childhood economic hardship, and adult economic hardship, compared to participants in the lowest quartile of education quality, those in the highest quartile had significantly better baseline cognitive performance (*β* = 0.09, *p* < .001). Quality of education did not consistently show an association with change in cognitive function over 4 years. Literacy and greater educational attainment were each independently associated with better cognitive function at baseline and less cognitive decline.

**Discussion and Implications:**

Quality of education, literacy, and years of education, while interrelated, also show independent associations with cognitive functioning in older Puerto Ricans. The downstream factors of literacy and years of education were more closely related to age-related cognitive decline than quality of education.

**Translational Significance**: This study provides additional support for the importance of both quality and quantity of education for cognitive function later in life. Few studies have examined historical indicators of education quality in relation to aging outcomes, and to our knowledge, none of them were in Hispanic/Latino populations. Because these early-life factors are modifiable, the potential translational and public health significance is high. In addition, because Puerto Rico is currently undergoing educational reforms related to the ongoing economic crisis and outward migration, findings can inform expected long-term consequences of changes in early-life education.

The population in Puerto Rico is rapidly aging, with the percentage of older adults aged 65+ years now 21.3%, which is higher than any U.S. state U.S. Census Bureau (2019). Caribbean Hispanics have been found to have relatively high incidence rates of Alzheimer’s disease and mild cognitive impairment (González et al., 2019; Tang et al., 2001; Vardarajan et al., 2015). Older Puerto Ricans, included in some studies under the term “Caribbean Hispanics,” also have a high prevalence of hypertension, diabetes, and low education levels (Pérez & Ailshire, 2017; Tucker et al., 2000). Despite Puerto Rico’s status as a commonwealth of the United States and Puerto Ricans’ increased risk for developing Alzheimer’s disease, this group is largely overlooked in aging studies (González et al., 2019).

The Puerto Rican Elderly: Health Conditions (PREHCO; Palloni et al., 2005) study was initiated in 2002 as the first population-based study of adults aged 60 and older on the island. Members of this cohort were born between the early 1900s and mid-1940s. There was no mandatory school attendance policy in Puerto Rico until the mid-1950s. For comparison, all states had compulsory schooling laws for children by 1918. The 1920s to early 1940s also marked a period of high unemployment on the island (McEniry et al., 2008; Robles & Benowitz, 2010). Many Puerto Ricans lived in rural areas and depended on the sugar cane industry for employment, which was seasonal, leaving many workers with limited income for half of the year (McEniry et al., 2008). During this same time of year, hurricane season brought hot and humid weather that facilitated the spread of infectious diseases, destruction to infrastructure, economic hardship, and high mortality rates (Government of Puerto Rico Department of Education, 1935; Robles & Benowitz, 2010).

The importance of education stems from its theorized role in brain development and its influence on cognitive functioning throughout the lifespan. Low levels of educational attainment have been found to predict cognitive function, cognitive impairment, and incident dementia in older adults (Glymour & Manly, 2008; Karp et al., 2004; Rogers et al., 2009; Sharp & Gatz, 2011). The most frequently cited mechanism to explain this relationship is the concept of cognitive reserve (Sharp & Gatz, 2011; Stern, 2012). Researchers hypothesize that education increases “cognitive reserve” that serves as a buffer for the clinical expression of brain pathology. Those with greater reserve are thought to have better functioning of brain networks or compensation for deficits that delay the appearance of clinical symptoms of dementia despite the presence of brain pathology due to Alzheimer’s disease or other forms of dementia (Tucker & Stern, 2011).

Education is especially relevant to understanding dementia disparities in diverse populations given the well-known disparities in educational attainment for many U.S. minority populations, as well as the potential variability in educational quality. The connection between more years of education and better late-life cognitive functioning has been examined extensively in previous research. For example, in one prominent study, Manly and colleagues (2004) found that among older African Americans, years of education was significantly associated with measures of verbal abstraction, phonemic fluency, and naming. Also, in a widely cited study of Catholic nuns, Mortimer and colleagues (2003) found that lower educational attainment was significantly associated with dementia.

Compared to research on years of education and cognitive outcomes, quality of education has been studied relatively little, and has relied mostly on measures such as self-rated literacy (Kavé et al., 2012), reading ability (Manly et al., 2002, 2004), vocabulary (Carvalho et al., 2015), or a mixture of reading ability, reading comprehension, writing, and self-rated English proficiency (Sisco et al., 2015). Less is known about the interrelationship of literacy, years of education, and extrinsic factors such as school funding, length of school year, and student–teacher ratio (Crowe et al., 2013; Sisco et al., 2015). Previous findings indicate that historical quality of education proxy measures is associated with level of cognitive performance but not change over time (Carvalho et al., 2015; Crowe et al., 2013; Sisco et al., 2015).

The role of quality of education in cognition among older Puerto Ricans may be particularly unique because of the history of U.S. influence on all aspects of life on the island and the overall less favorable socioeconomic conditions than the mainland. Approximately 38% of the population aged 65+ years in Puerto Rico is living below the poverty line, which is a much higher rate than in any U.S. state (Administration for Community Living, 2019). The current project examines whether years of education and indicators of education quality are associated with cognitive aging outcomes in a cohort of older adults in Puerto Rico, adding to the sparse existing research on early-life circumstances and late-life cognitive outcomes in diverse populations. We hypothesized that historical indicators of higher quality of education, literacy, and years of education would each be independently associated with baseline cognition at older age. However, we did not expect quality of education to be associated with rate of change in cognition based on previous research.

## Method

### Participants

**Figure 1.**
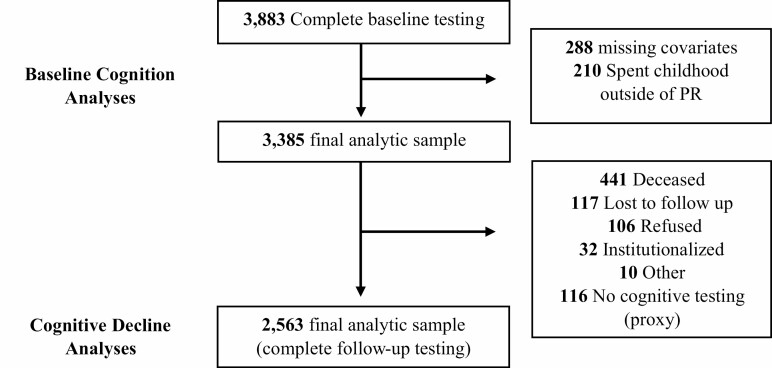
Analytic sample sizes for baseline cognitive function using baseline data from 2002 to 2003 and 4-year follow-up data (2007–2008) for cognitive decline analyses.

A sample of 4,291 community-dwelling adults aged 60+ years comprised the baseline target sample who agreed to participate in the PREHCO study in 2002–2003 (see Palloni et al., 2005). Interviews were conducted in participants’ homes at baseline and 4-year follow-up. Of the baseline participants, 408 were not interviewable, leaving a total of 3,883 participants with completed baseline cognitive testing (Figure 1). There were 210 participants excluded from the quality of education analyses because they reported spending their childhood outside Puerto Rico and could not be assigned Census or education data for Puerto Rican municipalities. At 4-year follow-up, 2,563 of the analytic sample completed cognitive testing. All study materials were orally administered by trained interviewers in Spanish. Participants were mainly recruited door-to-door based on census data and maps of the municipalities. The study was originally approved by the University of Puerto Rico Institutional Review Board (IRB) and University of Wisconsin IRB; the current project was additionally approved by the University of Alabama at Birmingham IRB.

### Measures

#### Cognitive function

The minimental Cabán (MMC) was used to measure global cognitive functioning at baseline and 4-year follow-up. The MMC was created to be more appropriate for use with Hispanic populations compared to the Spanish version of the Mini-Mental State Examination (MMSE), which is a simple translation to Spanish (Sánchez-Ayéndez et al., 2003). The MMC was found to have superior sensitivity and specificity compared to the Spanish translation of the MMSE in detecting clinically diagnosed dementia in a clinic-based sample of older adults in Puerto Rico (Sánchez-Ayéndez et al., 2003). The MMC is scored on a 0–20 scale, with higher scores indicating better cognitive performance. Similar to the MMSE, it includes measures of orientation, immediate and delayed verbal recall, copy of intersecting pentagons, and comprehension of a three-step command. However, the MMC also includes domains that are not in the MMSE, such as visual memory for a complex figure (viewing the figure for 15 s and then immediately drawing it from memory) and executive functioning (clock drawing and abstraction).

#### Quality of education

Two of our indicators of quality of education were chosen due to their availability in historical education reports during the time period when this cohort began schooling (1920–1940): school year length and student–teacher ratio. Participants began schooling in the mid-1930s, on average, with the entire cohort turning school age before 1950. Data were gathered from three reports ranging from 1926 to 1945 from the Commissioner of Education in Puerto Rico (Government of Puerto Rico Department of Education, 1928, 1935, 1947) and supplemented by data from the U.S. Census Bureau for 1935 (school attendance) and 1940 (literacy).

There is no one source in Puerto Rico with a complete collection of the Commissioner of Education reports from the 1920s through the 1940s, but we found that many were available from the University of Puerto Rico library system. Reports were chosen in order to have at least one report that included at least some information on indicators of quality from each decade during which the majority of participants attended school. A third of the sample was born before 1928, another third from 1928 to 1935, and the last third after 1935. Thus, we were able to identify three education reports with information relevant to education quality that also provided information separately for each municipality, corresponding to fiscal years of 1926–1927, 1933–1934, and 1945–1946.

There was inconsistency in the availability of data across the years 1920–1940, with no one year containing both length of school year and student–teacher ratio stratified by municipality. Therefore, we used information on either school year length (1926–1927; 1933–1934) or student–teacher ratio (1945–1946). Most of the Commissioner of Education reports during this time period either did not provide data by municipality or did not include measures of interest for educational quality. To supplement our data from education reports, we used Census data from 1935 for percentage of children aged 7–13 attending school for each municipality, as well as Census data from 1940 on the percentage of children 10 years or older who were able to read and write per municipality. Participants were then matched to these data based on report of the municipality he/she lived in the most before the age of 18, and whether they lived in an urban or rural area.

Three municipalities, Cataño, Canóvanas, and Florida, were not included in the 1926–1927 Commissioner of Education report because they did not become municipalities until 1927, 1970, and 1971, respectively. School system data were included from the previously established municipality names of Barros (Cataño), Loíza (Canóvanas), and Barceloneta (Florida). The Commissioner of Education report and Census reports starting in 1933 and forward include Cataño as an independent municipality.

School year length, student–teacher ratio, attendance, and literacy levels for each municipality were combined into one quality of education variable using principal component analysis. The highest loadings for the principal component were .85, .74, .70, and .62, for literacy levels in 1940, school attendance in 1935, school year length in 1926, and school year length in 1933, respectively. Because student–teacher ratio (1945) had a particularly low loading on the principal component, it was eliminated.

#### Literacy and years of education

Self-reported ability to read was measured with a yes/no question (asked by the interviewer). We coded years of education as the total number of years corresponding to level of educational attainment reported, then grouped as 0–3 years, 4–7 years, 8–11 years, 12 years, and 13+ years.

#### Childhood covariates

Similar to the Health and Retirement Study (HRS), items measuring childhood circumstances were collected retrospectively (Vable et al., 2017). Childhood economic hardship was measured by summing two items about participant’s general economic conditions in the household (i.e., “In general, would you say that the economic conditions in the household in which you grew up were good, average or bad?” and “Did you suffer economic hardship that prevented you from eating regularly, adequately clothing yourself or receiving the necessary medical attention?”), with scores ranging from 0 to 3 and higher scores equaling higher economic hardship. Participants also rated childhood health on a 5-point scale (excellent, very good, good, average, or poor), which has been previously used by the HRS (Elo, 1998). Higher scores on this measure reflect better health.

#### Adulthood economic hardship and health covariates

Adult economic hardship was measured by summing two variables regarding difficulty paying for daily living expenses (frequently, sometimes, or never) and difficulty paying for health care (frequently, sometimes, or never) with higher scores reflecting greater economic hardship. Scores ranged from 0 to 4. To measure vascular risk factors, a summed index score of vascular health conditions was calculated including self-reported diabetes, hypertension, myocardial infarction, congestive heart failure, and stroke/transient ischemic attack. Scores ranged from 0 to 5, with higher scores representing a greater number of vascular conditions. Depressive symptoms were assessed using a Spanish-language version of the 15-item Geriatric Depression Scale (Sheikh & Yesavage, 1986). This measure asks participants yes or no questions regarding depressive symptoms experienced within the last week. The Spanish version has previously been used in clinical settings at the University of Puerto Rico. Reliability analysis showed a Cronbach’s alpha of .86 and a factor analysis of items supported a one-factor solution with all items loading onto this first factor. Higher scores reflect greater depressive symptoms.

### Data Analysis

We initially examined sample descriptive characteristics and bivariate relationships between quality of education and variables of interest. Spearman’s correlations were used to examine bivariate associations between quality of education and ordinal or ratio variables of interest, and *t* tests were utilized for the association between educational quality and categorical variables. For the primary analyses, a series of regression models added variables sequentially, with demographic variables and quality of education entered first, followed by literacy and years of education, and then the additional childhood and adulthood covariates were included in a final model. Quartiles for quality of education were used to examine the possible dose–response association between quality and cognition. All data analyses were performed in SAS for windows, version 9.4 and IBM SPSS Statistics for windows, version 24.

## Results

Baseline sample characteristics are shown in Table 1. Age range at baseline for our sample was 60–102 years (mean age 72) and approximately 60% of this sample was female. Approximately half had less than an eighth grade education and 7.8% of participants reported being illiterate. For quality of education variables, the average number of school days in session was 183 days in 1926–1927 and 181 days in 1933–1934. The average classroom size was 54 students per teacher but reached up to 84 students in rural municipalities.

**Table 1.** Baseline Characteristics of Participants (*N* = 3,385)

Characteristic	*N* (%)	*M* (*SD*)	Range
Age		72 (8.27)	60–102
Sex (female)	2,025 (59.82)		
Days of school (1926)		183.56 (3.89)	174–190
Days of school (1933)		181.56 (4.86)	140.2–188.9
School attendance (1935), %		62.91 (7.02)	43.32–78.59
Literacy (1940), %		69.32 (6.54)	46.67–80.84
Student–teacher ratio (1945)		54.46 (6.03)	28–84
Education		8.13 (4.81)	0–18
Nonliterate	265 (7.83)		
Baseline cognition		16.61 (2.42)	0–20
Childhood economic hardship		1.44 (1.10)	0–3
Childhood health		3.45 (1.19)	1–5
Adult economic hardship		2.84 (1.30)	0–4
Vascular risk factors		1.21 (1.06)	0–5
Depressive symptoms		3.34 (3.42)	0–15

*Notes*: Depressive symptoms are measured by the 15-item Geriatric Depression Scale where a score >5 indicates depressive symptomatology. Percent literate is at the municipality level and is equal to the amount of people aged 10 years and older who could read and write reported by the Census. The variable nonliterate is self-reported literacy from the PREHCO questionnaire.

In bivariate analyses, the quality of education factor score was positively correlated with years of education and baseline cognition (Table 2). More years of education was also significantly associated with higher baseline cognition. Older age was related to lower education and lower baseline cognition.

**Table 2.** Correlations Among Early- and Late-Life Factors

Variable	1	2	3	4	5	6	7	8	9
1. Age	—								
2. Baseline cognition	−0.21**	—							
3. Quality of education	0.00	0.14**	—						
4. Years of education	−0.19**	0.30**	0.21**	—					
5. Childhood economic hardship	0.02	0.07**	0.09**	0.23**	—				
6. Childhood health	0.03	0.10**	0.10**	0.15**	0.27**	—			
7. Adult economic hardship	0.07**	0.06**	0.10**	0.20**	0.14**	0.13**	—		
8. Vascular risk factors	0.07**	−0.07**	−0.04*	−0.07**	−0.08**	−0.11**	−0.12**	—	
9. Depressive symptoms	0.06**	−0.16**	−0.08**	−0.17**	−0.17**	−0.22**	-0.25**	0.17**	—

*Notes*: Spearman’s correlation coefficients shown.

*p < .05. **p < .01.

Table 3 shows the association between baseline cognitive function and variables of interest. In the initial model controlling for age and sex, when compared to the lowest quartile of quality of education, the third and the fourth (highest) quartiles showed significantly higher baseline cognitive function. When years of education was controlled, only the highest quartile remained significantly related to baseline cognition. The results did not change in the fully adjusted model accounting for additional childhood and adulthood factors (Model 3). Fully adjusted results indicated that greater literacy was related to better cognitive function, and education groupings of 8–11 years, 12 years, and 13+ years (compared to 0–3 years) were all related to significantly higher baseline cognition. As expected, the highest education group (13+ years) showed the largest size of association with cognitive function.

**Table 3.** Education Variables Associated With Baseline Cognitive Function

	Model 1		Model 2		Model 3	
Variable	β	*SE*	β	*SE*	β	*SE*
Age	−0.21**	0.00	−0.17**	0.00	−0.17**	0.00
Sex (female)	0.04*	0.08	0.06**	0.08	0.07**	0.08
Quality of education (ref.: low)						
Rank 2	0.03	0.11	0.00	0.11	0.00	0.11
Rank 3	0.06**	0.11	0.03	0.11	0.02	0.11
High	0.17**	0.11	0.10**	0.11	0.09**	0.11
Literacy			0.11**	0.17	0.11**	0.17
Education (ref.: 0–3 years)						
4–7 years			0.04	0.13	0.04	0.13
8–11 years			0.14**	0.14	0.13**	0.14
12 years			0.15**	0.14	0.14**	0.15
13+ years			0.23**	0.14	0.22**	0.14
Childhood economic hardship					−0.02	0.04
Childhood health					0.05**	0.03
Adult economic hardship					−0.02	0.03
Vascular risk factors					−0.03	0.04
Depressive symptoms					−0.10**	0.01

*Notes*: The lowest years of education, 0–3 years, serves as the reference group. For the quality of education composite, the lowest quartile of quality serves as the reference group.

*p < .05. **p < .01.

Results from linear regression models examining the association between education variables and change in cognition are shown in Table 4. Being in the highest quartile in terms of quality of education emerged as significantly related to more cognitive decline at follow-up, but only when years of education and literacy were included in the same model. Both literacy and more years of education were significantly associated with less cognitive decline. Having 13+ years of education had the strongest association with reduced cognitive decline over the 4-year follow-up.

**Table 4.** Education Variables Associated With Cognitive Decline

	Model 1		Model 2		Model 3	
Variable	β	*SE*	β	*SE*	β	*SE*
Age	0.29**	0.01	0.27**	0.01	0.27**	0.01
Sex (female)	0.00	0.11	−0.01	0.10	−0.02	0.10
Baseline cognition	0.44**	0.02	0.52**	0.02	0.52**	0.02
Quality of education (ref.: low)						
Rank 2	0.00	0.14	0.02	0.14	0.02	0.14
Rank 3	−0.01	0.14	0.02	0.14	0.02	0.14
High	0.00	0.15	0.06**	0.14	0.06**	0.14
Literacy			−0.10**	0.22	−0.10**	0.22
Education (ref.: 0–3 years)						
4–7 years			−0.03	0.16	−0.04	0.17
8–11 years			−0.07**	0.18	−0.08**	0.18
12 years			−0.11**	0.19	−0.12**	0.19
13+ years			−0.23**	0.18	−0.24**	0.19
Childhood economic hardship					0.04*	0.05
Childhood health					0.01	0.04
Adult economic hardship					0.00	0.04
Vascular risk factors					0.03*	0.05
Depressive symptoms					0.02	0.02

*Notes*: The lowest years of education, 0–3 years, serves as the reference group. For the quality of education composite, the lowest quartile of quality serves as the reference group.

*p < .05. **p < .01.

## Discussion

Using a representative sample of older Puerto Rican older adults, we found that childhood quality of education was positively related to both years of education and to baseline cognitive functioning in late life. The results generally fit with past research on the relationship between quality of education and adult health and cognition from samples based elsewhere (Brenowitz et al., 2020; Crowe et al., 2013; Garcy & Berliner, 2018; Sisco et al., 2015). Past studies have similarly found that indicators of quality of education are more strongly associated with cross-sectional cognitive performance than change in cognition over time (Carvalho et al., 2015; Crowe et al., 2013; Sisco et al., 2015). However, after adjusting for literacy and years of education, there was a significant association between high education quality and more cognitive decline. While counterintuitive, this finding may align with the cognitive reserve theory. For example, it has been found that individuals with greater occupational attainment may have steeper declines after the initial appearance of clinical symptoms of dementia (Stern, 2012). However, we were unable to address this possibility directly in the current study due to lack of dementia ascertainment.

In terms of context for the current older adult sample compared to mainland U.S. samples, quality of education for this cohort may be expected to be more homogenous within municipalities because there were not segregated schools, and also because, unlike Puerto Rico, most states have multiple school districts within counties that lead to more heterogeneous educational policies. The Puerto Rico Department of Education is one of only four state/territory-wide public education systems in the United States. Despite these factors that would presumably lower variability in quality, we still identified significant links between education quality and cognitive performance in older adults.

It is also important to note that the structure and curriculum of schools at the time of our cohort’s schooling was very different from modern-day education systems in the United States. For example, in the 1930s, schools implemented a double enrollment strategy in which 57% of the total student body at this time attended school for half a day, with one session in the morning and a second session in the afternoon (Government of Puerto Rico Department of Education, 1935). This particular cohort of individuals was likely influenced by the economic changes during the early 1900s and the rapid changes in the education system on the island. Before 1940, the average educational attainment of the adult population was 2.7 years. Over the subsequent decades, education steadily increased, climbing to 8.7 years in the 1980s (similar to education levels seen in our sample, who on average would have been in their 50s during this decade), and then to 11th grade in 2000. Ladd and Rivera-Batiz (2006) state that Puerto Rico had one of the strongest educational development trajectories in the world during this time period.

While our study had many strengths, including the population-based sample and use of historical indicators of education quality, findings must be viewed considering several limitations. In terms of measures, performance-based reading ability would have been preferable to our self-reported literacy measure but was not included in this study. Also, an in-depth neuropsychological battery and clinical dementia diagnosis were not available. Another limitation, for educational quality: we were unable to find consistent data on variables such as school year length and student–teacher ratio broken down by municipality in the historic education reports. We also do not know which participants attended private schools, and data from the education reports applied only to public school systems. Students who attended private schools may have received better quality of education and stayed in school longer compared to public schools. While private schooling in Puerto Rico has steadily increased in recent decades, only 4.1% of elementary children were attending private schools in the 1940s and, therefore, represent a small percentage of the total number of children attending school during the time period of interest for our sample (Ladd & Rivera-Batiz, 2006). An additional issue is that questions used to measure economic hardship in childhood were retrospective and subjective. This is a common practice in life course epidemiologic research with older adults given the rarity of longitudinal studies from childhood through older adulthood. Results using subjective childhood variables should be viewed in light of potential recall and social desirability biases.

In summary, our results suggest that, in this representative sample of older Puerto Ricans born between the early 1900s and mid-1940s, indicators of education, including better quality, higher literacy, and more years, are related to better cognition in late life. Education in general appears to have an important influence on long-term health and cognition in older Puerto Rican adults. It may be that through better quality of education and more years of education, people built greater cognitive reserve that helped maintain normal cognitive function into older adulthood. Favorable indicators of education may also have provided more employment opportunities that lead to improved economic conditions, including access to health care. Greater access to health care, which may come with better education, may reduce prevalence of cardiovascular conditions that are associated with cognition (Beydoun et al., 2014). Individuals with higher education are also more likely to engage in cognitively stimulating activities throughout the lifespan that are thought to be protective against dementia (Beydoun et al., 2014; Ngandu et al., 2007; Richards & Deary, 2005).

Our findings are relevant to public policy in light of the current changes in the public education system in Puerto Rico. Puerto Rico closed hundreds of schools both before and after the hurricane due to lack of funding followed by structural damage to schools (Hinojosa et al., 2019). There has also been a decline in school enrollment due to families migrating to the U.S. mainland, a trend exacerbated by the hurricane (Trines, 2018). Future projections for the educational system include more public school closures that could result in overcrowding in some areas, as well as the privatization of school systems, which is likely to result in more variability in education quality (Trines, 2018). The restructuring of the Puerto Rican school system may have negative implications for the quality of education children receive and, in turn, long-term consequences in terms of aging and health.
